# Influence of Social Media on the Decision to Undergo a Cosmetic Procedure

**DOI:** 10.1097/GOX.0000000000002333

**Published:** 2019-08-08

**Authors:** Khalid Arab, Omar Barasain, Abdullah Altaweel, Jawaher Alkhayyal, Lulwah Alshiha, Rana Barasain, Rania Alessa, Hayfaa Alshaalan

**Affiliations:** From the * Department of Plastic and Reconstructive Surgery, King Saud University, Riyadh, Saudi Arabia; † College of Medicine, King Saud University, Riyadh, Saudi Arabia.

## Abstract

There is a rise of cosmetic procedures in Saudi Arabia. Social media have become a popular means of advertisements for cosmetic professional providers. There are currently no studies that determine the relationship between viewing cosmetic surgery advertisements and considering undergoing cosmetic procedures in female university students in Saudi Arabia.

**Methods:**

A cross-sectional study was conducted using an online survey. This survey determined the use of social media, cosmetic-related accounts followed by the respondent, and attitudes toward advertising for cosmetic treatments on social media. It also determines the influence of these accounts on female university students to consider undergoing cosmetic treatments in the future. The survey was distributed on multiple social media platforms and by Email.

**Results:**

Out of 816 completed questionnaires, 48.5% reported being influenced by social media to consider undergoing cosmetic procedures. Respondents (51.4%) follow plastic surgeons on social media (*P* < 0.001). The most common reported number of hours of social media use per day was >5 hours per day (53.2%; *P* < 0.026). Statistical significance was considered in relation to reporting being influenced by social media to consider undergoing cosmetic procedures in the future.

**Conclusions:**

Our findings revealed that viewing cosmetic surgery–related material on social media, spending longer hours on social media platforms, and having negative self-views when viewing social media are associated with an increased likelihood of considering undergoing cosmetic procedures in the future. Future studies using a validated questionnaire that assesses the likelihood of being influenced by social media to undergo cosmetic treatments are encouraged.

## Abstract 2

Supplemental Digital Content is available in the text.

## INTRODUCTION

According to an article published by the *Australian Journal of Plastic Surgery*, cosmetic surgery is defined as any invasive procedure where the primary intention is to achieve what the patient perceives to be a more desirable appearance and where the procedure involves changes to bodily features that have a normal appearance on presentation to the doctor.^1^ Cosmetic treatments can be surgical, such as breast augmentations and rhinoplasty, and nonsurgical such as botulinum toxin and fillers. Social media are gaining popularity worldwide as being a method used to advertise for cosmetic treatments. Social media are integrating into many aspects of daily life in Saudi Arabia, which has one of the most active populations on social media platforms. It is important to determine the role of these advertisements in influencing the patient’s decision to undergo a cosmetic procedure.

Social media platforms are used as methods of communication, education, entertainment, promotion, and lifestyle aspects, such as diet, fashion, and specifically beauty. Beauty used to be advertised almost exclusively through television shows and commercials, but now it has a large presence in advertisements done by social media influencers. A Forbes article shows that a review from a trusted social media figure is the equivalent of a word-of-mouth relay.^17^ In general, there is a lack of studies conducted to determine the factors that influence the decision of the patient to undergo a cosmetic treatment after viewing these advertisements. Also, to the knowledge of the authors, there has yet to be a validated questionnaire that might elicit this effect.

The most popular social media applications among Saudis are as follows: Twitter, Instagram, Snapchat, and WhatsApp. According to a study published in 2017, there are over 12 million active Facebook users and >53% active Twitter users in Saudi Arabia, making it the country with the highest Twitter penetration worldwide. Other platforms such as Instagram and Path are extremely popular in Saudi Arabia compared with other parts of the world.^3^

Studies have previously suggested a relation between time spent on social media and body dissatisfaction.^4^ This relation can sometimes be attributed to face distortion in pictures, especially noted in “selfies” due to the close proximity of the cameras.^4^ In this study, we focus on social media and their effect of promoting cosmetic procedures. The advertisements are done either directly or indirectly through self-promotion of clinics and fashion influencer–driven promotion, respectively. Twitter is a popular means of advertising for cosmetic surgery. It is considered the best platform to disseminate information regarding plastic surgery and public education^5^ as an analysis of the phrase “Plastic Surgery” and hashtag “#PlasticSurgery” showed (43.8% of tweets contained professional information and resources).^6^ A study conducted on Arabic-speaking adults concluded that the most influential factors in choosing a treating physician regarding personal features were their reputation.^7^

Even though the Saudi community is considered conservative, plastic surgery is becoming one of the fastest growing industries in the region, with the emphasis on its cosmetic branch, offering minimally invasive solutions to appearance-related issues the patients may have. Although cosmetic surgery has always been popular among the older population, some studies suggest that its popularity is particularly rising among young female university students in Saudi Arabia.^7,8^ The literature review shows evidence of the effect of social media on undergoing a cosmetic procedure. There is a limited scope of studies conducted on female Saudi university students with regards to their attitude toward cosmetic treatments. Thus, to the best of our knowledge, there are no studies conducted in Saudi Arabia which target the effect of social media on undergoing cosmetic procedures. This study aims to evaluate the influence of viewing social media advertisements related to cosmetic surgery, following plastic surgeons on social media, and other important related factors on the intention of young Saudi females to undergo cosmetic treatments in the future.

## MATERIALS AND METHODOLOGY

We conducted a quantitative observational cross-sectional study. We used convenience sampling. The study population consisted of female students in universities in the city of Riyadh, Saudi Arabia. The inclusion criteria for the participants were as follows: female university students currently studying in Riyadh, Saudi Arabia, 18–30 years old. The sample consisted of both. The exclusion criteria were as follows: non-Saudi nationality. A sample size of 375 was calculated using a 95% CI and a 0.05 degrees of freedom and was increased to improve the overall precision of the study. A final sample size of 816 female students was obtained. The questionnaire was constructed using Survey Monkey and distributed online among the target population via Email, Twitter, and WhatsApp over a period of 4 months. Entries were collected and data were extracted for statistical analysis in SPSS version 22.0 (Armonk, NY: IBM Corp).

The respondents have been fully informed about the goals and purposes of the study and have participated voluntarily and they were able to leave the study at any given time. An informed consent was required at the beginning of the electronic survey. The study was approved by the Institutional Review Board committee in King Saud University on May 14, 2018, with the assigned project number E-18–3223.

After extensive review of the literature related to the topic, a questionnaire consisting of 36 questions was developed in 2018 under the supervision of a consultant plastic surgeon currently practicing in the field of cosmetic surgery in Saudi Arabia. The questionnaire demonstrated the demographic characteristics of the participants, previous history of cosmetic treatments among the participants, use of social media and specific accounts being followed by the participant, related psychosocial effects of viewing specific social media content, the attitudes of the participants toward the use of social media for cosmetic surgery purposes, and the influence of social media on their decision to undergo a cosmetic procedure (SDC1, http://links.lww.com/PRSGO/B138)

## RESULTS

### Section I: Demographics

A total of 1,018 surveys were started, and 925 were completed. After excluding non-Saudi participants, the total was 816 responses. Statistical significance was considered in relation to considering undergoing cosmetic procedures after viewing social media cosmetic advertisements, and a *P* value of <0.05 was considered to be statistically significant.

The mean age of the respondents in this survey was 21.15 ± 2.52. They were mostly from public universities, and of non–health-related specialties (n = 372, 45.5%), with more than half of them (n = 436, 53.4%) reporting a family monthly income of >20,000 SAR (Saudi Arabian Riyals) (5,000 USD) (Table 1).

**Table 1.** Influence of Social Media to Undergo Cosmetic Procedures

	N (%)
1. Have social media cosmetic treatment advertisements influenced you to consider undergoing a cosmetic treatment	
A. Yes	396 (48.5)
B. No	421 (51.5)
2. If cosmetic treatment advertisements on social media influenced you, which type of cosmetic treatment are you considering undergoing?	
A. Surgical procedures (liposuction, breast augmentation, rhinoplasty, etc)	74 (18.7)
B. Nonsurgical procedures (botulinum toxin, fillers, etc)	280 (70.7)
C. Both	42 (10.6)

### Section II: Influence of Social Media on the Decision to Undergo Cosmetic Procedures

The study showed that 48.5% (n = 396) of respondents were influenced by advertisements to undergo cosmetic treatments. Of them, two thirds considered undergoing nonsurgical procedures (botulinum toxin, fillers, etc) (n = 280, 70.7%), and only 18.7% would consider undergoing surgical procedures due to social media influence.

### Section III: Personal and Family History of Cosmetic Treatment

**Fig. 1.**
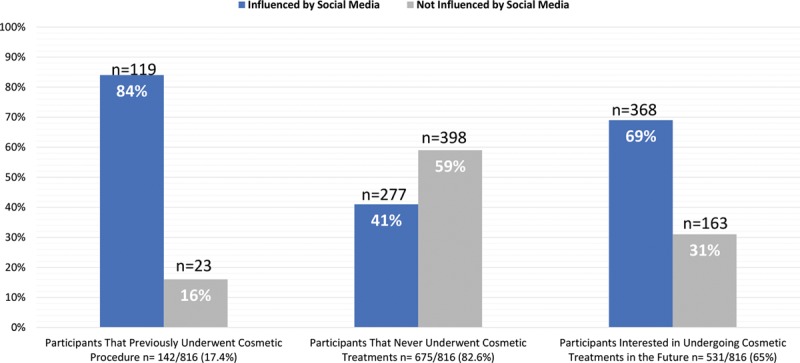
Participants’ personal and family history of cosmetic treatments.

Approximately a quarter of those who were influenced by social media’s cosmetic advertisements considered undergoing a cosmetic procedure in the future (183, 22.4%), whereas 14.6% (119) of them have already received cosmetic treatment of some form in the past. The results also show that the respondents have more friends that have undergone cosmetic procedures (60.2%) than family members (41.6%) (Fig. 1).

All the aforementioned results were statistically significant (*P* < 0.001).

### Section IV: General Use of Social Media

**Fig. 2.**
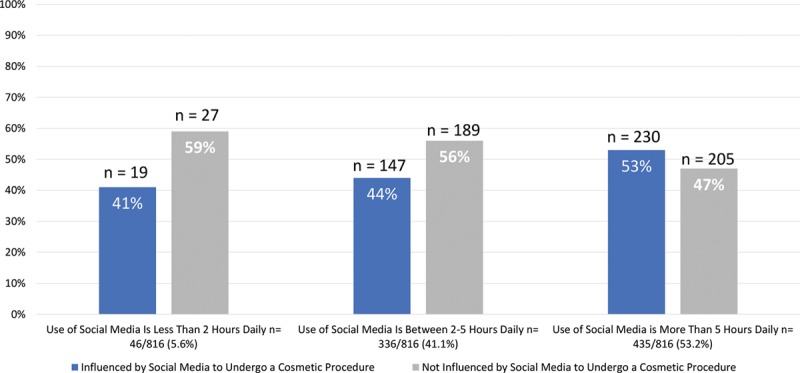
Number of daily hours spent on social media platforms by participants.

**Fig. 3.**
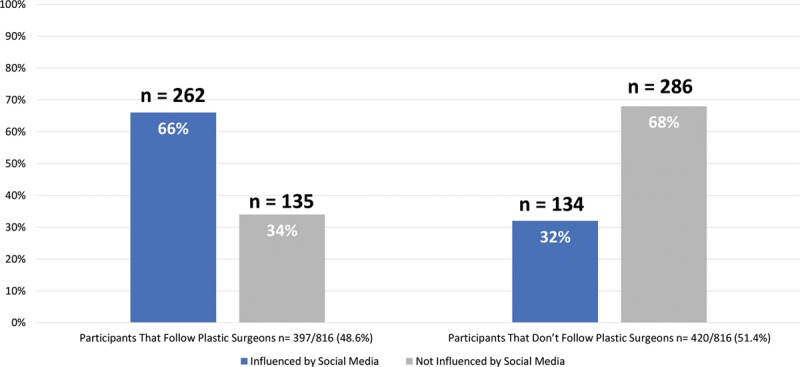
Following plastic surgeons on social media platforms.

**Fig. 4.**
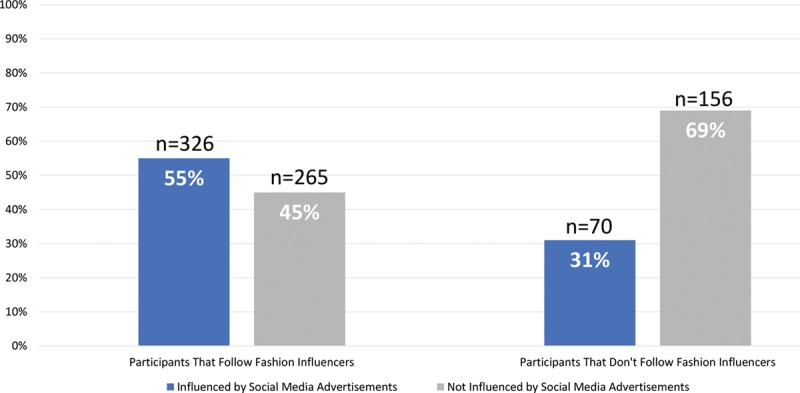
Following fashion influencers on social media platforms.

The results show that participants spending 5 hours or more daily on social media (53.2%) were among the highest percentage of participants that reported being influenced by advertisements. Snapchat had the highest level of influence among social media platforms. Despite the influence of social media, 51.9% still prefer using websites such as Google as a source of information on cosmetic procedures. Fashion influencers surpassed both plastic surgeons and beauty tip accounts in follower percentage (72.3%). More than half of the viewers (455, 55.7%) reported that fashion influencers who they follow advertise for cosmetic procedures (Table 2 and Figs. 2–4).

**Table 2.** General Use of Social Media

	N (%)
What is the social media application you use the most?	
A. Snapchat	215 (26.3)
B. Twitter	206 (25.2)
C. Whatsapp	160 (19.6)
D. Instagram	116 (14.2)
E. YouTube	77 (9.4)
F. Others	43 (5.3)

Respondents (57.6%) follow social media accounts that provide beauty tips, and 51.4% follow social media accounts of plastic surgeons. Entertainment and education were the main reasons behind following these accounts. The study shows that a Saudi female student who follows a plastic surgeon on social media is more attracted and influenced by cosmetic advertisements (32.1%), which is double the percentage of those who do not follow surgeons (16.4%). The influence of ads is also similar in families who follow fashion influencers or celebrities advertising cosmetic treatment.

### Section V: Attitudes Toward Advertisements for Cosmetic Procedures on Social Media

**Fig. 5.**
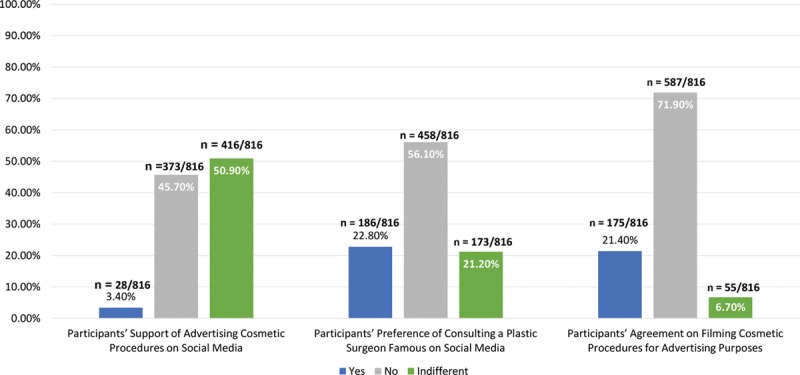
Attitude toward advertisements for cosmetic procedures.

Although it was previously stated that half of the respondents follow plastic surgeons, only 22.8% prefer consulting a plastic surgeon who is famous on social media, and only 23.4% inquired about a plastic surgeon’s degrees and qualifications (Fig. 5). Equal proportion of participants thought that cosmetics clinics would be more appealing if they were active on social media and those who do not think so (31.7% and 32.7%, respectively; *P* < 0.212).

Regarding the filming of procedures, about half of the respondents (48.5%) agreed with filming of a patient’s cosmetic procedure only for educational purposes, whereas 21.4% agreed with using those videos for advertisement purposes.

The vast majority of respondents did not, however, agree to filming their own cosmetic procedures and posting it on social media. Only 9.9% would allow it if they got benefits such as discounts, offers, etc.

The percentage of respondents who were not influenced by cosmetics advertisements is higher in those who would not allow filming which is 72%, and it is also higher in the respondents who were against using social media to advertise cosmetic procedures (45.7%).

In regards to the main purpose of using social media in advertisement of cosmetic surgeries, 39.4% believe that it is to promote cosmetic specialists and clinics, followed by demonstrating cosmetic surgery results (32.8%; *P* < 0.015).

### Section VI: Psychosocial Effects of Viewing Cosmetic-Related Material on Social Media

The results demonstrate how the respondents’ sense of self-esteem and attractiveness is affected by social media personalities. A quarter of the respondents constantly compared their appearances to social media celebrities and influencers, and 29% (*P* < 0.001) felt unattractive when doing so, yet only 21.5% (*P* < 0.001) reported that they would feel happier if they looked more like those celebrities. Only 18% (*P* < 0.001) would seriously consider undergoing a cosmetic procedure to improve their social media status, and a particularly low percentage of 6.4% (*P* < 0.001) would actually consider undergoing a cosmetic procedure if this procedure was especially popular among social media influencers. The remaining two thirds, 60% (*P* < 0.001) of the respondents, did not feel less attractive and did not feel a reduced self-esteem. Participants (42.8%; *P* < 0.001) felt uncomfortable posting their pictures on social media without using filters, equaling the percentage that feels comfortable with doing so (42.6%; *P* < 0.001). The influence of cosmetic treatment ads is indeed higher in those who felt uncomfortable posting their pictures without filters (26.1%; *P* < 0.001) in comparison to those who were did not share that sentiment (16.4%; *P* < 0.001).

Regarding the respondents’ friends and community, 23.9% of respondents routinely compared their pictures to others, and as a result, 20.3% felt pressured to change their appearance to look better on social media. Corresponding with the effect of social media influencers, only 10.4% said that they may consider pursuing cosmetic treatment if it was popular among their peers.

Finally, most participants did not feel that the society describes females as “unattractive” if they do not receive any cosmetic treatment (751, 91.9%; *P* < 0.001).

## DISCUSSION

This study aimed to determine the effect of social media on female university students’ willingness to undergo cosmetic treatments because there are no studies that measure the influence of social media on undergoing cosmetic procedures.

The main findings of the study were that almost half of the respondents were influenced by advertisements in their decision to undergo cosmetic treatments. Also, two thirds of respondents were either possibly or surely interested in undergoing cosmetic procedures in the future, and more than half of the respondents follow plastic surgeons and fashion influencers that talk about cosmetic procedures. Moreover, more than a quarter constantly compare their appearances to social media’s celebrities and feel unattractive when doing so.

The results showed that social media cosmetics treatment advertisements might have had an influence on the consideration of undergoing cosmetic treatments. The majority of those considering undergoing cosmetic treatments due to the effect of advertisements preferred nonsurgical procedures.

The study also shows that those who were influenced by cosmetic ads usually had family members or friends who have also undergone cosmetic treatments.

Approximately a third of the respondents who reported being influenced by the advertisements followed plastic surgeons on social media. This may be explained by the preference of expert explanation of the procedures’ techniques and the illustration of the advantages and disadvantages of the procedures from a medical point of view.

With regards to the psychosocial effect of cosmetic-related viewing of social media, the greater part of the respondents is either possibly or surely interested in undergoing cosmetic procedures in the future, yet most of them do not consider them either because people they personally know or social media celebrities who they follow are undergoing them. Perhaps the underlying reason for females asking for cosmetic consultations is the pressure exerted by social media and by their peers, and not the fact that they actually want to change their appearance, or to change it in a way that makes them look unique and different than others. This coincides with the results of a study done by Furnham and Levitas,^4^ which revealed that people with low perceived self-attractiveness were the most willing to undergo cosmetic surgeries, and this might confirm the effect of low self-esteem on their decisions regarding cosmetic treatment. A study conducted in Taif University that assessed the knowledge, attitudes, and practices of cosmetic surgery among 220 female students found that although none of them had reported undergoing any cosmetic procedures, 79.1% had heard about cosmetic surgery from mass media. This relates to the findings of our study that a large percentage of the Saudi female university student population follow cosmetic surgery–related accounts on social media.^8^ Another study conducted at King Abdulaziz University among 600 Saudi female university students revealed that 2.2% had received cosmetic surgery and that 23.3% of respondents agreed that mass media affected their decision to undergo cosmetic procedures, also supporting the findings of our study.^7^ Our study also showed a larger prevalence of cosmetic procedures in respondents (17.5%) than both previous studies conducted in Saudi Arabia.^7,8^

About a third of the respondents felt that cosmetic surgery clinics which are active on social media seemed more appealing to them, which may be because of the clear and possibly glorified display of the services provided. Almost a third of the study population preferred consulting plastic surgeons who are famous on social media; this may be owing to the excellent reputation of the doctor as advertised by different influencers or by hearing the patient’s recommendations. Additionally, the study showed that close to half of the population is against advertising cosmetic surgeries on social media platforms; this might be due to belief that clinics would prioritize monetization over the quality of patient care.

The present study has several limitations. First, convenience sampling technique was used instead of random sampling, justified by its easy availability and accessibility to participants. Furthermore, the sample was only taken from female university students in Riyadh 18–30 years old and did not include other age groups nor did it include other regions in Saudi Arabia. For future studies, we recommend including different age groups such as middle-aged adults and expanding the study to other major cities in Saudi Arabia. Finally, regarding the data collected, it was self-reported, which might introduce response bias due to the effects of social desirability or selective memory. However, the questionnaire incorporated several aspects of the desired subject, which made it less likely that the results were remarkably affected by those biases. In addition, the main point of strength in this study is that it is the first of its kind which focused on the effect of social media on cosmetic procedures with such elaboration and diversity, and we believe that the chosen study population accurately represents the group that regularly uses social media for the purposes observed by this study.

## CONCLUSIONS

This study aimed to determine the effect of social media on undergoing cosmetic procedures. Our findings revealed that participants who reported viewing cosmetic surgery–related material on social media, spending longer hours on social media platforms, and having negative self-views when viewing social media, reported in higher cases that they consider undergoing cosmetic procedures in the future than the participants who answered differently. Future studies using a validated questionnaire that assesses the likelihood of being influenced by social media for undergoing cosmetic treatments are encouraged.
